# Failure analysis of bolts in deluge valve bonnet in cooling tower system in petrochemical plant

**DOI:** 10.1038/s41598-025-94743-2

**Published:** 2025-04-23

**Authors:** M. A. El-Zomor, M. H. Ahmed, F. S. Ahmed, M. A. Elhelaly

**Affiliations:** Tabbin Institute for Metallurgical Studies (TIMS), Helwan 109, Cairo, 11421 Egypt

**Keywords:** TWIP steel, Fractography, Cold deformation, Stress corrosion cracking, Engineering, Materials science

## Abstract

Machine bolts installed on the bonnet of OS&Y (outside screw & yoke) deluge gate valve 6″, were found cracked or broken in service. The gate valves are part of the petrochemical plant fire protection system. The result of chemical analysis of the received bolts samples, indicates that their composition corresponds to a TWIP steel, having high manganese (16 wt.%) and high chromium (9 wt.%). The microstructural examination of cross sections of bolts, revealed an austenite microstructure with high number density of deformation twinning and slip lines specially at the failure zones, which is typical of the cold deformed TWIP steels. The hardness distribution within the bolt’s head and the head to shank zone was not homogeneous, ranging between 225 HV_0.1_ in the shank central zone and 470 HV_0.1_ at the curved highly stressed zone of the bolt neck. Both the cracked and broken bolts appeared corroded specially at the failure zone of the head to shank neck. The EDS analysis of corrosion products inside cracks indicated high concentration of Cr, Mn, Cu, O and traces of mineral elements of Si, S, Cl, K and Ca. The XRD analysis identified the Chromium Iron Manganese intermetallic Cr_5_Fe_6_Mn_8_ and Iron Manganese Carbide Fe_2.7_Mn_3_C in the bolt matrix and formation of “Spinel” Copper Chromite CuCr_2_O_4_ and Iron Manganese Oxide MnFe_2_O_4_. The cracks appeared to originate at corrosion pits formed at the crevice between the bolt head and the underneath washer and nut at the head to shank fillet radius and on bolt neck zone, which have high concentration of corrosive species from the humid salty marine atmosphere. The cracks then propagate in perpendicular direction to the tensile stress direction of the bolt torque. The fractography examination of the cracked bolts cross sections and the broken bolts fracture surface depicted mixed ductile/dimpled mode with areas of quasi-cleavage transgranular and intergranular crack propagation. These observations suggest the failure mechanism to be hydrogen embrittlement–assisted chloride—stress corrosion cracking HE-assisted Cl-SCC.

## Introduction

This study aims at investigating the failure locations and modes of failure in bolts installed on the bonnet of outside screw & yoke (OS&Y) deluge gate valve 6″ that were found cracked or broken in service. Deluge valve is known as a system control valve in a deluge system, used for fast application of water in a spray system. Deluge valve protects areas such as power transformer installation, storage tank, conveyor protection and other industrial applications^1^. A bolt is a threaded fastener utilized throughout industry to secure two or more mechanical parts together. There are a wide variety of threaded fasteners available including hex head bolts, carriage bolts, machine screws, studs, tapping screws, socket head bolts and plow bolts. Bolt failure has been the attributed cause of several accidents in mechanical systems and structures. Due to the different use environment and requirements of bolts, there are various forms of bolt fracture failure. Petrescu et al.^2^ predicted the failure behavior of several types of steel under different conditions by the simulation of the fastener manufacturing process. Meanwhile, two types of defects have been reported during the bolt forming process. The first one is the external crack due to the exhaustion of material ductility. For instance, shear cracks are generally initiated nearby the bolt head region during the bolt forming process due to stress concentrations^2,3^. The second one is the internal crack caused by adiabatic shear band phenomenon^4–6^. Maruschak et al.^7,8^ evaluating fracture patterns of polycrystalline materials based on the parameter analysis of ductile separation dimples and fatigue striations. El-Zomor et al.^9,10^ studied the failure of Mn-steel and SS by corrosion fatigue. Although several studies on bolts have been conducted using plain carbon steels, stainless steels, and Al alloys, none have reported on the bolt forming process for TWIP steels. The bolt forming features of the TWIP steel were different from those of the conventional low-strength metals due to their higher strain hardening rate and their unique plastic deformation mechanism of deformation twining^11^. Also, high-strength bolts can be fabricated from TWIP steels in the absence of heat treatment, which is not widely practiced. Therefore, this study focused on identifying the causes of failure of the TWIP steel bolts.

**Fig. 1:**
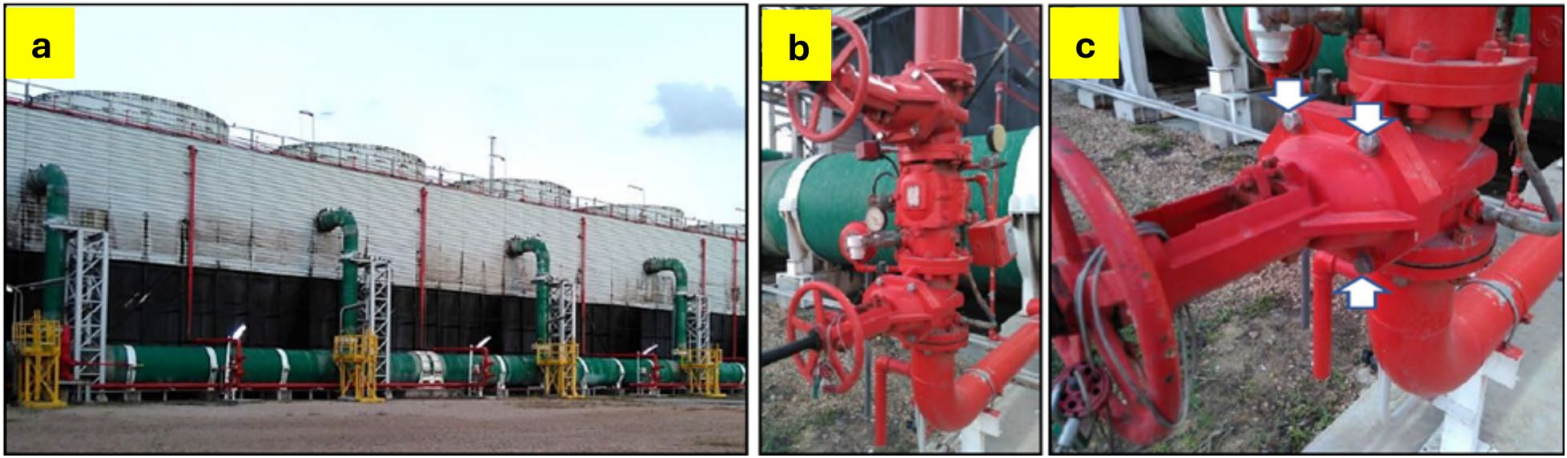
(**a**) The station units of the fire protection system supplied by the same OS&Y valve vendor. (**b**) The deluge gate upper (U) and lower (L) valves in one of the stations. (**c**) the location of the 4 bonnet bolts (arrowed).

In our case, machine bolts installed on the bonnet of outside screw & yoke (OS&Y) deluge gate valve 6″ were found cracked or broken. The gate valves are part of the plant fire protection system are shown in Fig. 1 and the service conditions are illustrated in Table 1.

**Table 1:** Service conditions.

Operation data	
Bolt and valve (bonnet) material	
Bolts, nuts and washers	Not available
Valve	ASTM A126 Class B gray cast iron
Cooling tower water analysis	
Almost all measurements are within specs limits, Cl^–^	Close to the upper limit of 300 ppm
Na^+^	200 – 250 ppm
Working pressure	9.3 brag
Service temperature	Ambient temperature 5 °C to 40 °C
Valve type / model	OS &Y Gate valve 6″ ANSI 150, Resilient wedge seat
Operating service	continuous
Number of bolts on the valve Bonnet	4 bolts
Stresses	Not available

## Investigation of failed bolts

### Visual examination

**Fig. 2:**
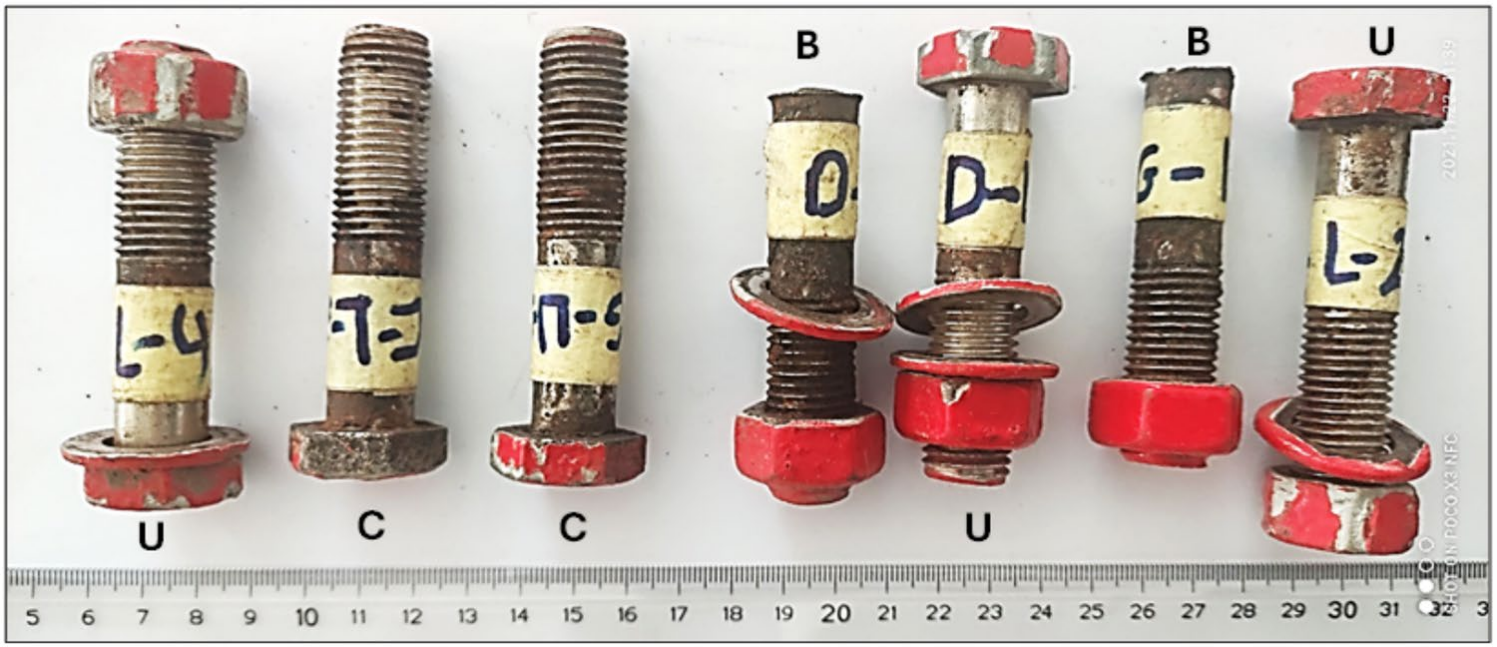
The received cracked and broken bolts samples.

**Fig. 3:**
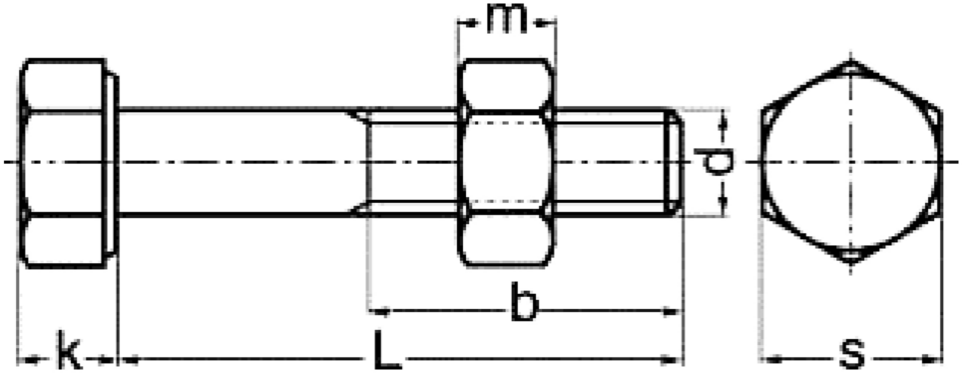
Bolt dimensions: **k** (Head Length): 8 mm, **S** (Across Flat): 23 mm, **L** (Nominal Length): 72 mm, **b** (Thread Length): 38 mm and **d** (Bolt Diameter): 13 mm.

The received seven bolt samples are shown in Fig. 2. All heads, washers and nuts are covered with red paint, while the shanks are covered with a layer of dark brown rust, which is caused by moisture from environment surrounding the bolts. The bolts dimensions are shown in Fig. 3.

**Fig. 4:**
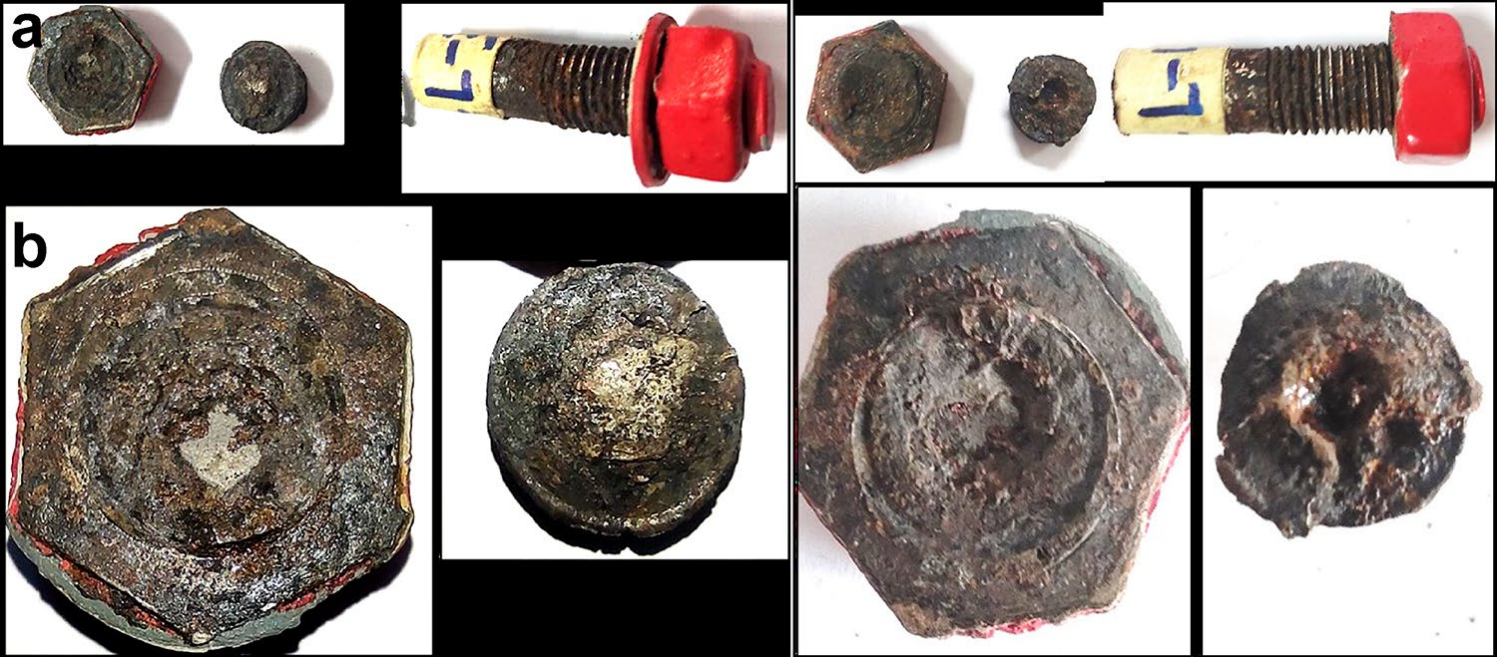
(**a**) The broken bolt samples, (**b**) Closeup view of the head side fracture surface and the mating shank side fracture surface, showing ductile cup (head) and cone (shank) fracture appearance.

**Fig. 5:**
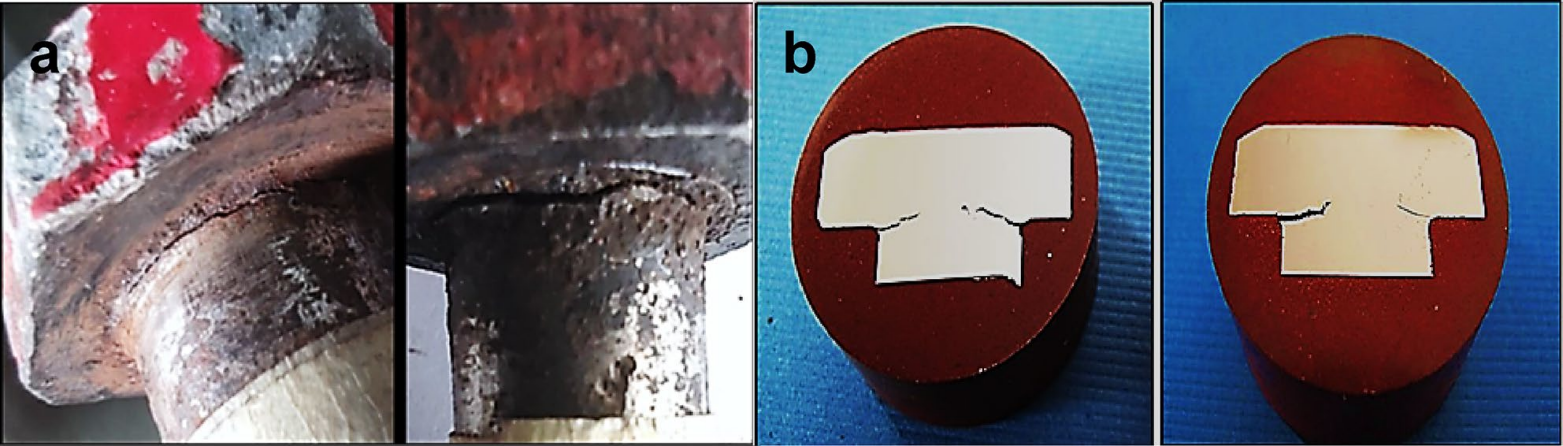
(**a**): two bolts are cracked at the head to shank position. (**b**): The longitudinal cross-sections from cracked bolts through heads mounted in Bakelite.

Two of the samples are broken at location of head to shank fillet radius B, two are cracked C at the same location and three are apparently un-broken **U**, as shown in Fig. 2. Figure 4 shows the broken bolt samples, along with a close-up view of the head-side fracture surface and the mating shank-side fracture surface. The image highlights the ductile cup (head) and cone (shank) fracture appearance. This photograph illustrates a crack that initiated on the outer surface and propagated toward the center of the bolt bar. Figure 5a shows two bolts with cracks at the head-to-shank position, while Fig. 5b displays the cracked bolts cut longitudinally through the heads and mounted in Bakelite, revealing cracks encircling the bolt heads and propagating nearly perpendicular to the torquing tensile stress.

**Fig. 6:**
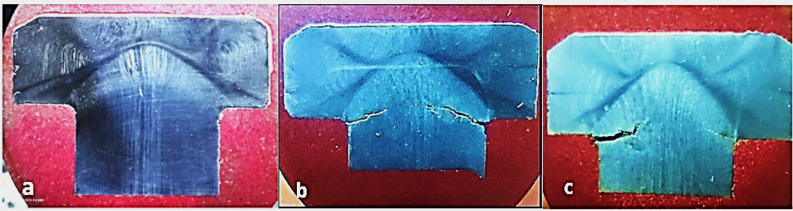
Closeup view of an etched macrograph of longitudinal cross section through (**a**) the head of unbroken bolt showing the material flow lines. (**b, c**) showing cracks initiating at the *head to shank fillet radius*, which is the most stressed zone between the bolt head and shank.

The excessive transverse cracking, occurring at an angle of 20° to 30°, indicates the presence of a torsional stress effect. Closeup view in Fig. 6 shows an etched macrograph of longitudinal cross section through (a) the head of unbroken bolt revealing the material flow lines. The flow lines ran parallel to one another along the longitudinal direction of the bolt in the central area below the head; while the flow lines in the head top and sides conformed to the cold formed shape of the head profile. Figure 6 (b, c) is showing cracks initiating at the head to shank fillet radius, which is the most stressed zone between the bolt head and shank. Tiny microcracks are seen branching from the main crack. The wide crack in Fig. 6c path indicates the effect of corrosion and suggests possible over-torque during installation.

### Chemical analysis of the bolt material

The material of the received ferrules was analyzed with the aid of ARL™ 3460 Optical Emission Spectrometer unit and the chemical analysis is given in the following Table 2 in weight percentages**.**

**Table 2:** Chemical composition of TWIP steel in wt.%.

Element	C	Mn	Si	P	S	Cr	Cu	Fe
Wt.%	**0.035**	**15.8**	**0.24**	**0.06**	**0.005**	**9.10**	**1.70**	**Bal**

The result of chemical analysis indicates that the composition of the received *failed bolts* material is high manganese, high chromium TWIP (Twin Induced Plasticity) Steel, which is a category of the AHSS (Advanced High Strength Steel) group.

The result of chemical analysis of the received new (unused) bolts showed also the same chemical composition.

### Chemical analysis of the washers and nuts

The nuts and washers were also analyzed by EDS technique, which showed TWIP steel composition similar to that of bolts having high Mn and Cr contents.

- Nut: 14 wt.% Mn – 11 wt.% Cr.
- Washer: 14 wt.% Mn – 13 wt.% Cr.

### Light microscopy

**Fig. 7:**
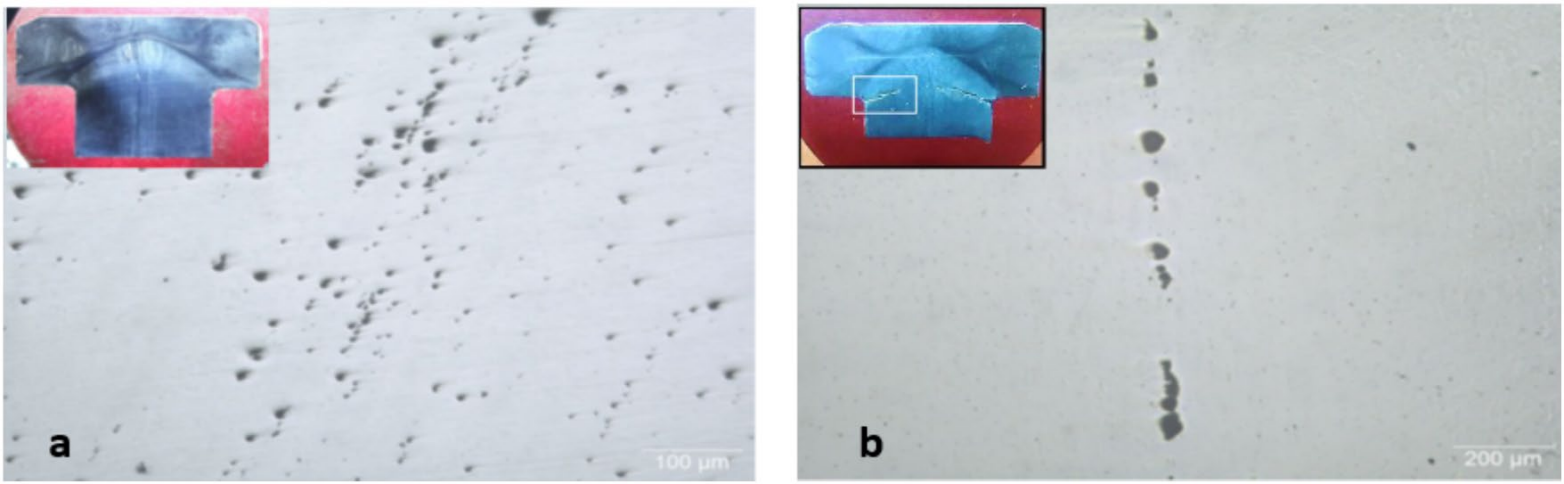
Light micrograph of the as polished longitudinal cross section of (**a**) the unbroken bolt, which is showing clusters of non-metallic inclusions. (**b**) unbroken bolt, which is showing train of almost interconnected non-metallic inclusions.

**Fig. 8:**
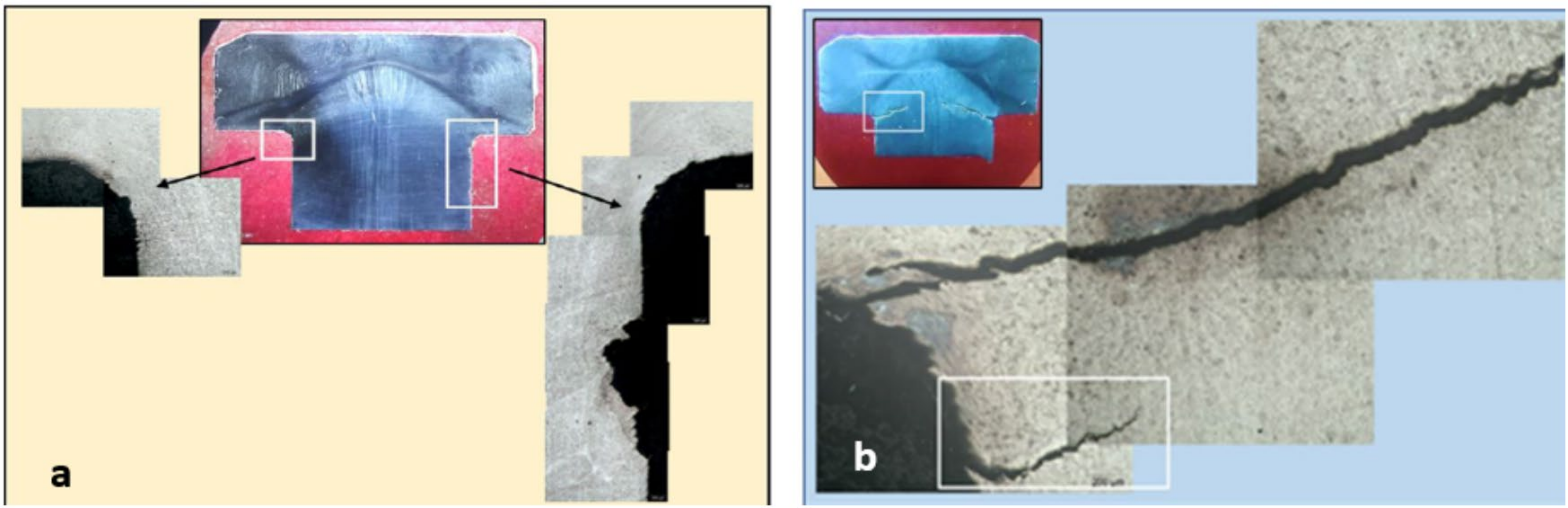
Low magnification composed light micrographs of cross section of (**a**) the unbroken bolt, showing corrosion pitting at the head-to-shank fillet radius and on the bolts. (**b**) the cracked bolt, showing multiple cracking at the highly stressed location of the bolt’s head to shank fillet radius.

**Fig. 9:**
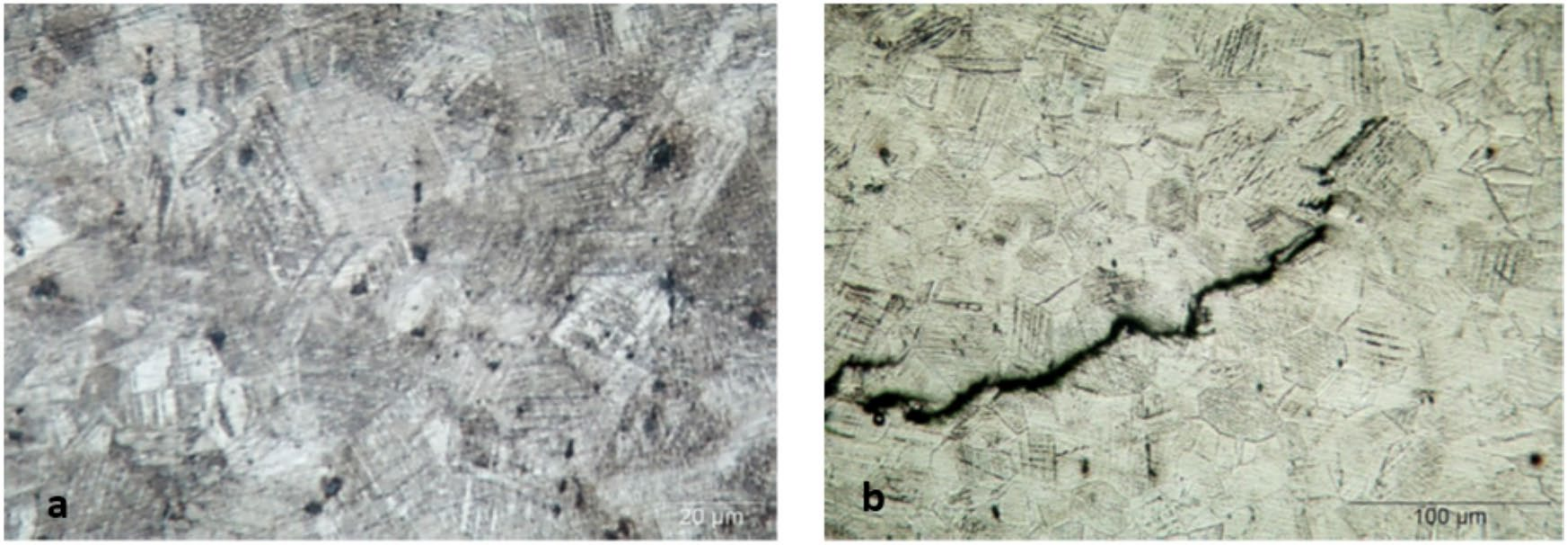
Light micrograph of an etched cross section of (**a**) the unbroken bolt, showing austenitic microstructure with high number density of deformation twinning and slip lines, typical of TWIP steel. (**b**) the broken bolt, showing the crack end at higher magnification, depicting high number density of deformation twins indicating highly stressed zone.

**Fig. 10:**
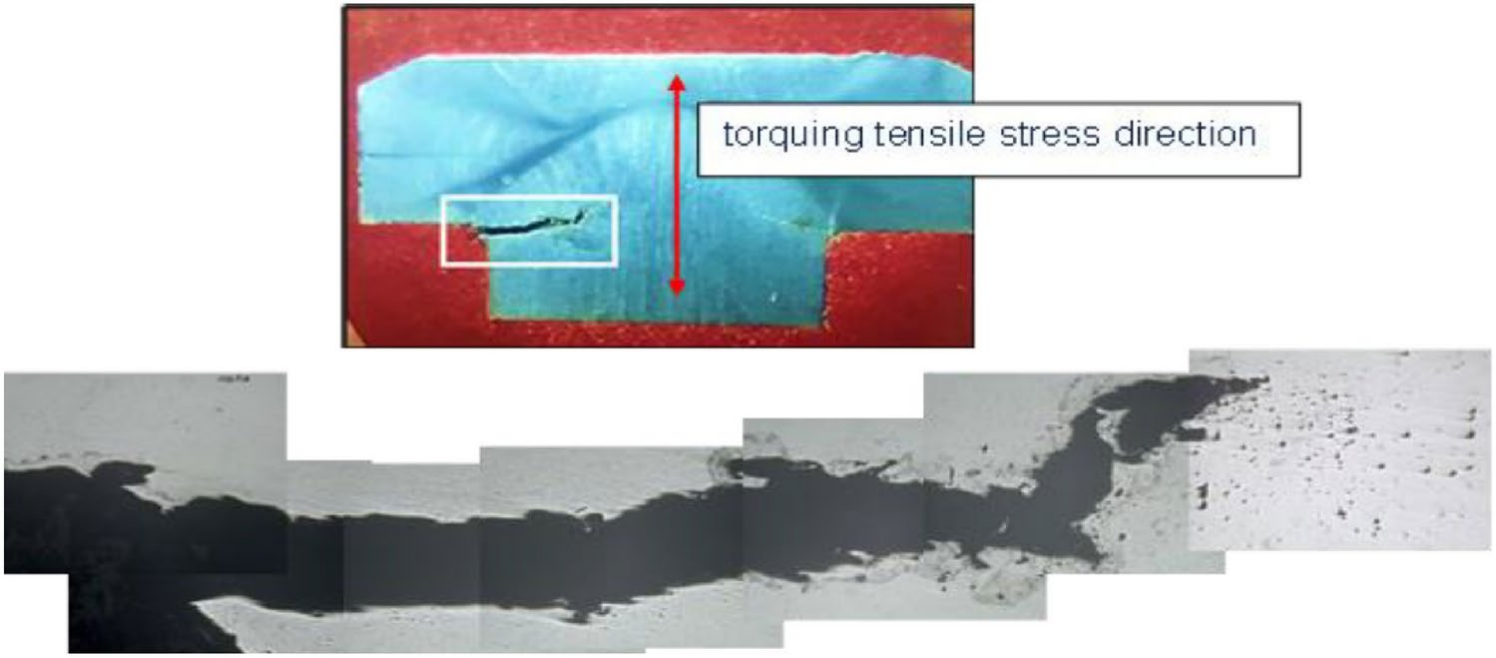
Low magnification composed light micrographs of cross section of the cracked bolt, showing wide crack starting at corrosion pit on the head to shank fillet radius location and propagating perpendicular to the torquing tensile stress direction, indicating the effect of corrosion and possible over torquing of bolts during installation.

Cross sections from the received unbroken (U) and cracked (C) bolt samples were cut longitudinally across the bolt head using water-cooled cut-off machine and prepared by standard metallographic methods for examination using metallurgical light microscope with digital camera. The sections were etched with *Kaling reagent* (Water 57.54%, Ethyl Alcohol 23.89%, hydrochloric acid 14.41%, cupric chloride dihydrate 1.65%, isopropyl alcohol 1.32%, methyl alcohol 1.19%). Light micrograph of the as polished longitudinal cross section of unbroken bolt, Fig. 7 (a), is showing clusters of non-metallic inclusions. Some of the non-metallic inclusions appear arranged in almost interconnected trains, which may facilitate propagation of cracks through the bolt material, and the others are littering the matrix and in the Fig. 7 (b) is also showing a train of almost interconnected non-metallic inclusions. The low magnification composed light micrograph of cross section of the unbroken bolt, Fig. 8 (a), is showing corrosion pitting at the head-to-shank fillet radius and on the bolt’s neck, indicated in the white rectangles on the middle macrograph. The deep corrosion pits are formed due to accumulation of corrosion species of the humid salty atmosphere in the crevice between the bolt head and the underneath washer in addition to the galvanic corrosion of the less noble cast iron valve body. The cracked bolt, shown in Fig. 8 (b), reveals multiple cracking at the highly stressed location of the bolt’s head to shank fillet radius. Figure 9 shows light micrograph of an etched cross section (Kaling reagent) of Fig. 9 (a) the unbroken bolt, depicting austenitic microstructure with high number density of deformation twinning and slip lines, typical of TWIP steel and Fig. 9 (b) the broken bolt, showing the crack end at higher magnification, where the observed high number density of deformation twins is indicating highly stressed zone. The zigzag trans-granular propagation path of the crack is a sign of possible hydrogen induced cracking effect. The low magnification composed light micrographs of cross section of the cracked bolt, Fig. 10, is showing wide crack starting at corrosion pit on the head to shank fillet radius location and propagating perpendicular to the torquing tensile stress direction, indicating the effect of corrosion and possible over torquing of bolts during installation.

### Hardness and tensile testing

A microhardness tester type *LECO LM 700* was utilized to measure the microhardness of the bolts samples cross sections applying load of 100 gf and dwell time of 10 s.

**Fig. 11:**
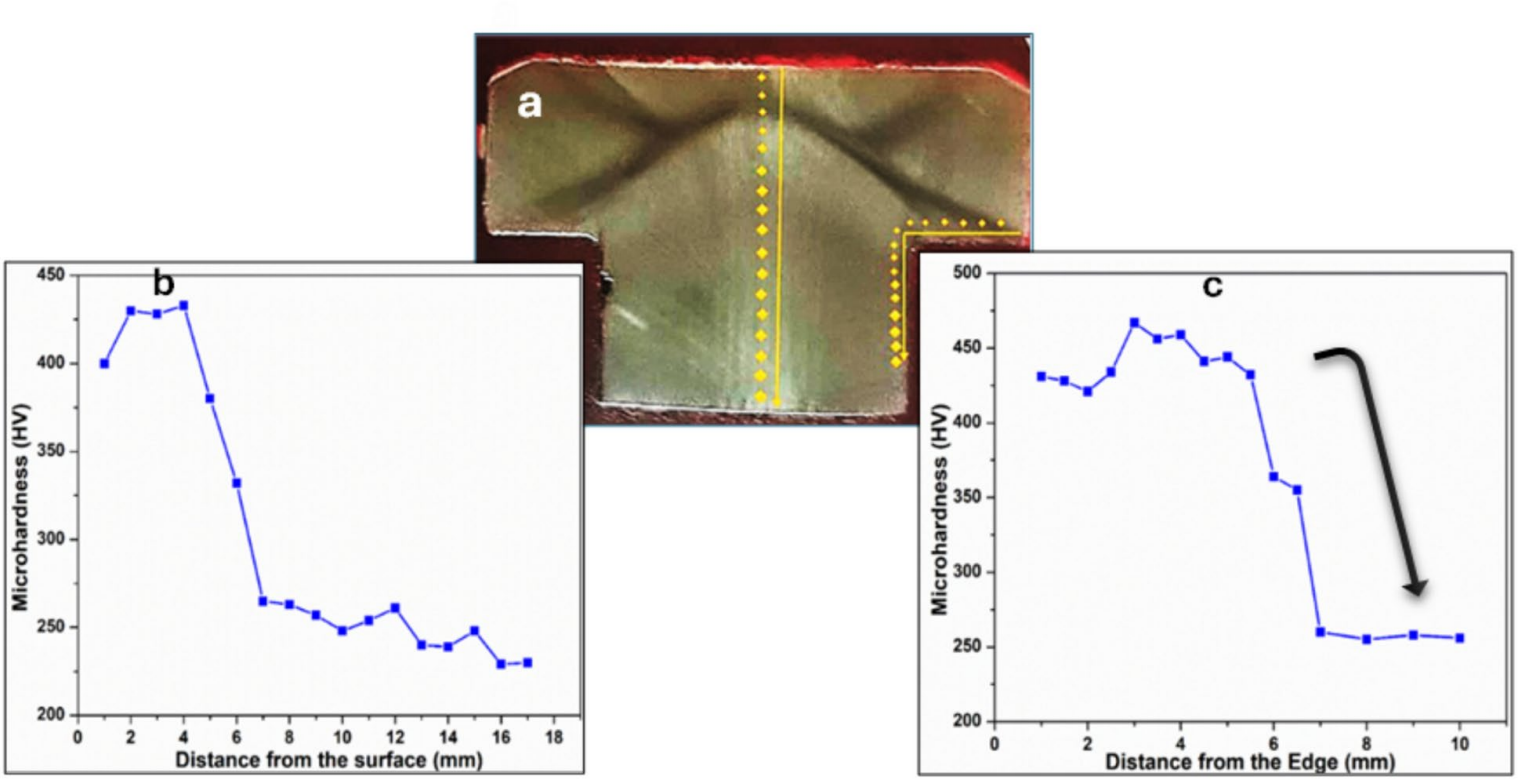
(**a**) Light macrograph of longitudinal cross section of the unbroken bolt sample, showing locations of microhardness indentations (**b**) Hardness number distribution across the bolt central longitudinal axis, (**c**) Hardness number distribution across the bolt head to shank fillet radius position.

The microhardness indentations at the central longitudinal axis and at the head to shank fillet radius are shown in the macrograph of cross section of Fig. 11(a). The corresponding hardness numbers across the bolt central longitudinal axis is shown in the profile curve of Fig. 11b, indicating high hardness of ~ 435 HV_0.1_ at the heavy cold-deformed part of the bolt head surface and decreasing to ~ 225 HV_0.1_ in the shank central zone, the deviation in hardness of the TWIP steel bolt with area was approximately 210 HV_0.1_. The hardness number distribution across the bolt head to shank fillet radius position is shown in profile curve of Fig. 11c with highest hardness values of ~ 470 HV_0.1_ at the high stress concentration area of the bolt neck radius and decreasing to the hardness value level of ~ 250 HV_0.1_ in the shank zone ~ 4 mm away from the head. The deviation in hardness along the fillet radius of the bolted TWIP steel with area was approximately 220 HV_0.1_. The tensile test is an essential method for assessing the mechanical properties of a TWIP steel bolt under uniaxial tension. This test provides critical parameters such as yield strength (514 MPa) and ultimate tensile strength (690 MPa). These values fall within the minimum range for TWIP steel.

### Scanning electron microscopy and energy dispersive spectroscopy

Scanning electron microscope *SEM INSPECT S50* was utilized for the examination of the cracked and fractured bolts cross sections and fracture surfaces to reveal the fine details of the microstructure at high resolutions and magnifications. The chemical composition of the non-metallic inclusions, corrosion scales and phase constituents were analyzed by Energy Dispersive X-Ray Spectroscopic analysis *EDS* point, line scan and X-ray mapping techniques using *BRUKERS EDS* analyzer attached to the *INSPECT S50* scanning electron microscope.

**Fig. 12:**
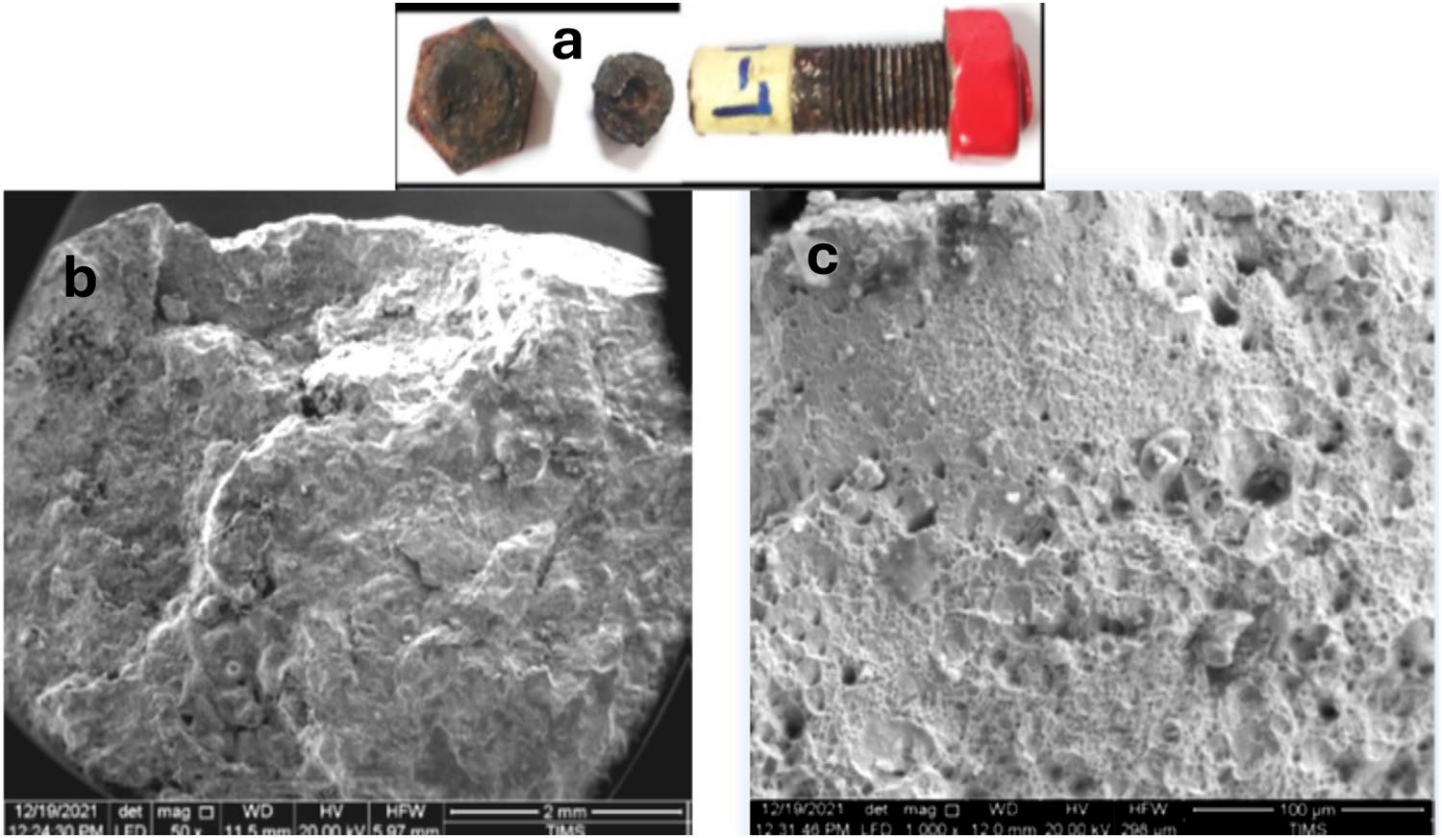
(**a**) The top macrograph is showing typical cup (head) to cone (shank) ductile fracture appearance of the bolt sample. (**b**) The low magnification SEM micrograph is showing part of the head fracture surface. (**c**) SEM micrograph is showing typical ductile dimpled fracture.

**Fig. 13:**
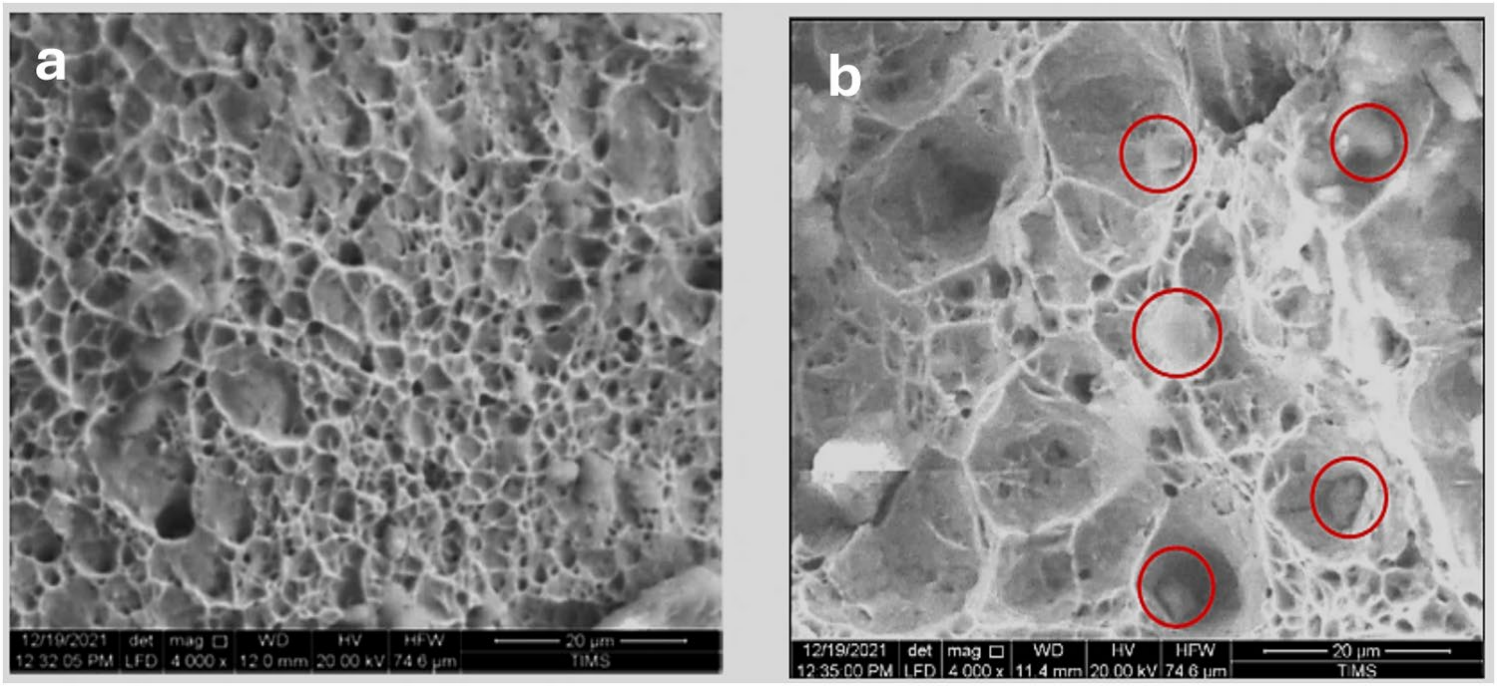
SEM micrographs of the fracture surface of the bolt. (**a**)**:** showing mixed dimpled and quasi-cleavage fracture appearance. (**b**)**:** another area showing dominant quasi-cleavage fracture around non-metallic inclusions (red encircled) indicating high hardness and highly stressed structure.

**Fig. 14:**
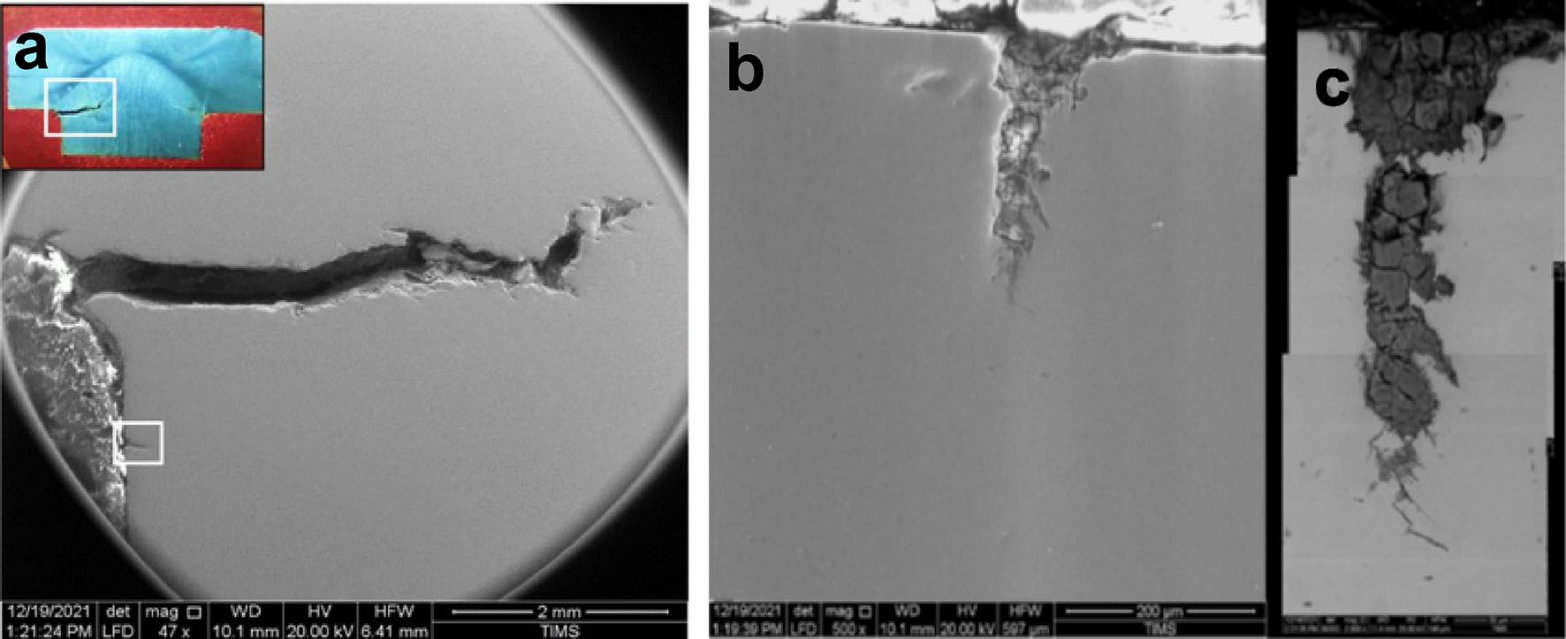
(**a**) SEM micrograph of a cross section of the cracked bolt showing wide crack at the bolt neck originating at corrosion pit with branching tiny cracks. (**b**)**:** SEM micrograph of the small crack (in white rectangle) originating at surface corrosion pit. (**c**)**:** SEM is a composed back scattered electron micrograph showing segregation of heavier material (dark grey) inside the crack.

**Fig. 15:**
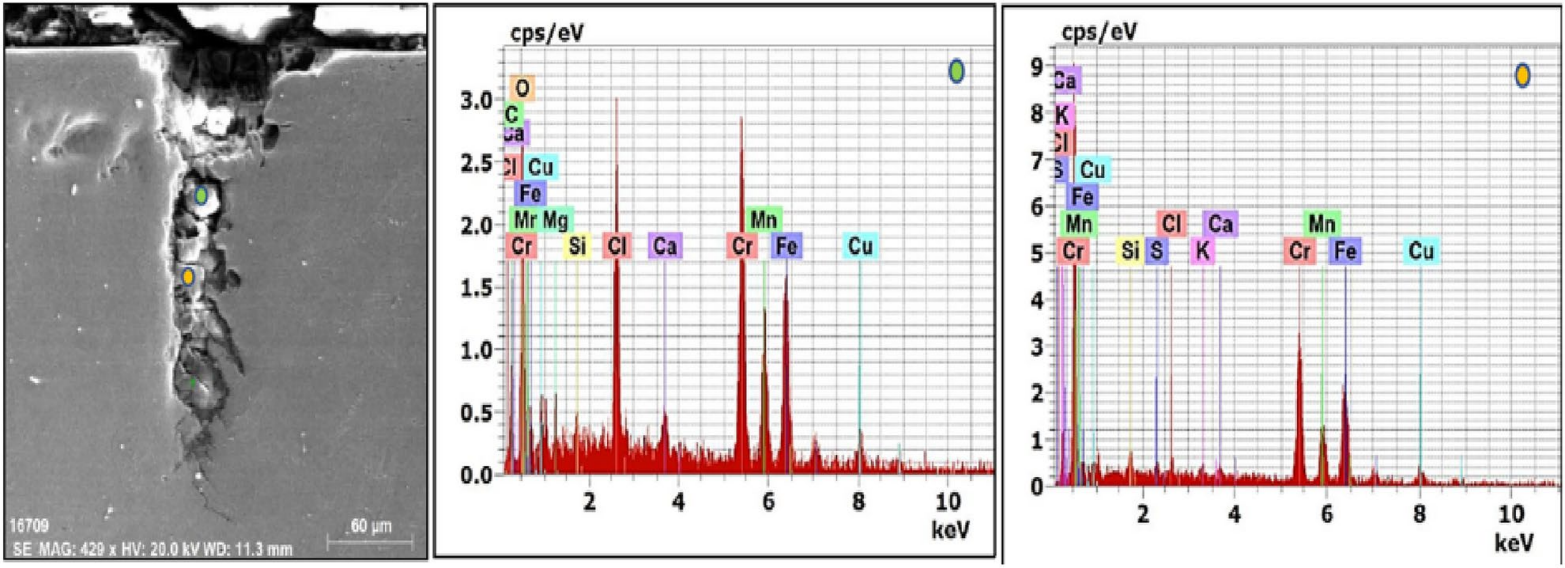
SEM micrograph, EDS spectrum and point analysis in the cracked bolt at orange point, showing high Cr (36.55 wt.%), high Mn (11.39 wt.%), high Cu (8,46 wt.%) and traces of mineral elements of Si, S, Cl, K and Ca. and at green point showing high Cl, C and O contents**.**

**Fig. 16:**
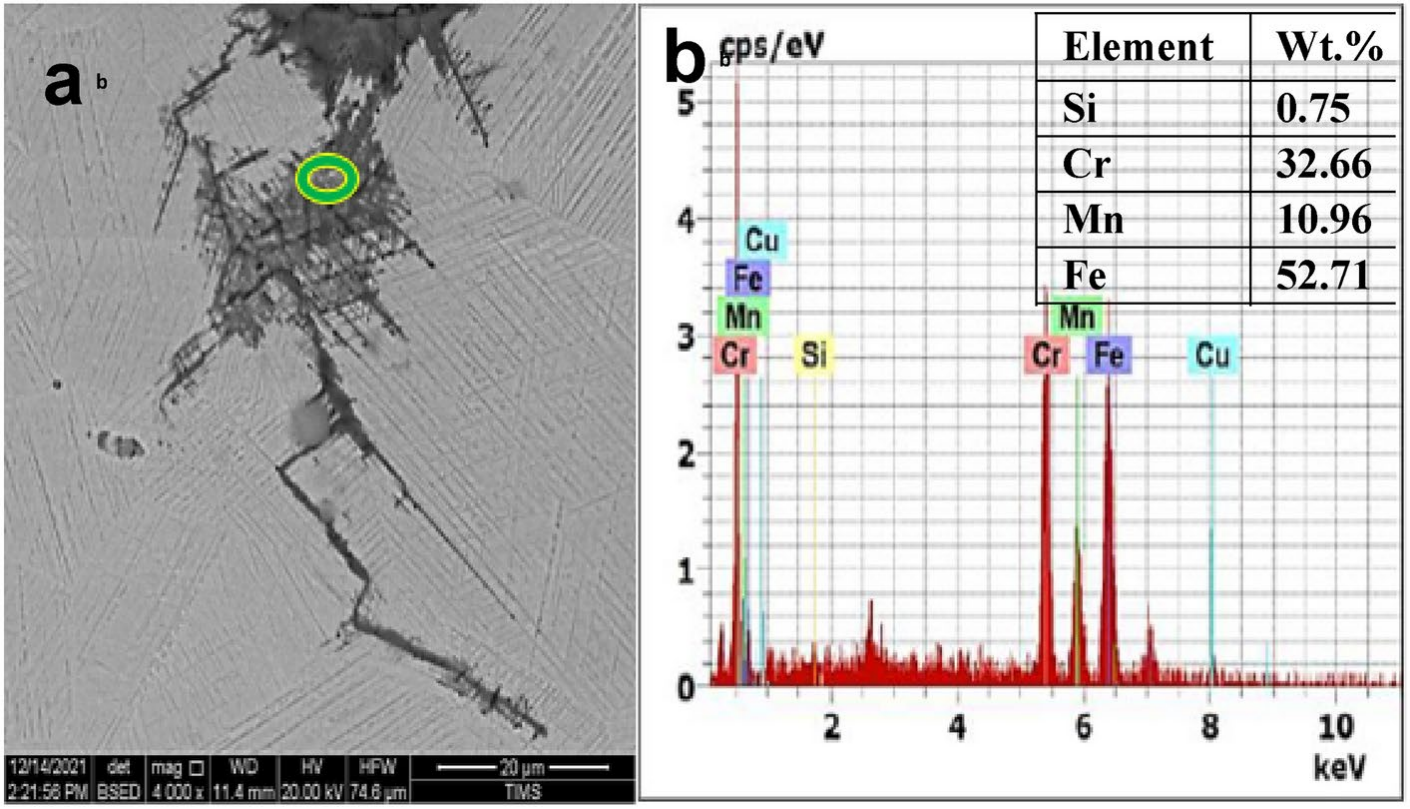
(**a**) SEM back scattered electron micrograph of the crack tip shown above depicting the penetration of the heavy element into the grain and twin boundaries, facilitating propagation of micro-cracking. (**b**) EDS spectrum and point analysis in cracked bolt at green point showing high Cr concentration (32 wt.%) inside the bottom of the wide crack.

The results of the SEM examination and EDS analyses are reviewed in the following figures (Figs. 12,13,14,15,16):

### X-ray diffraction analysis

A diffractometer unit type BRUCKER – D8 ADVANCE was used for XRD analysis of corrosion scale on the bolt fracture surface to identify the phases present in corrosion. Products and in cross section of bulk material applying Cu-target, at 40 kV / 40 mA. The results of the XRD analysis of surface scale are shown in the diffractograms of the following figures.

**Fig. 17:**
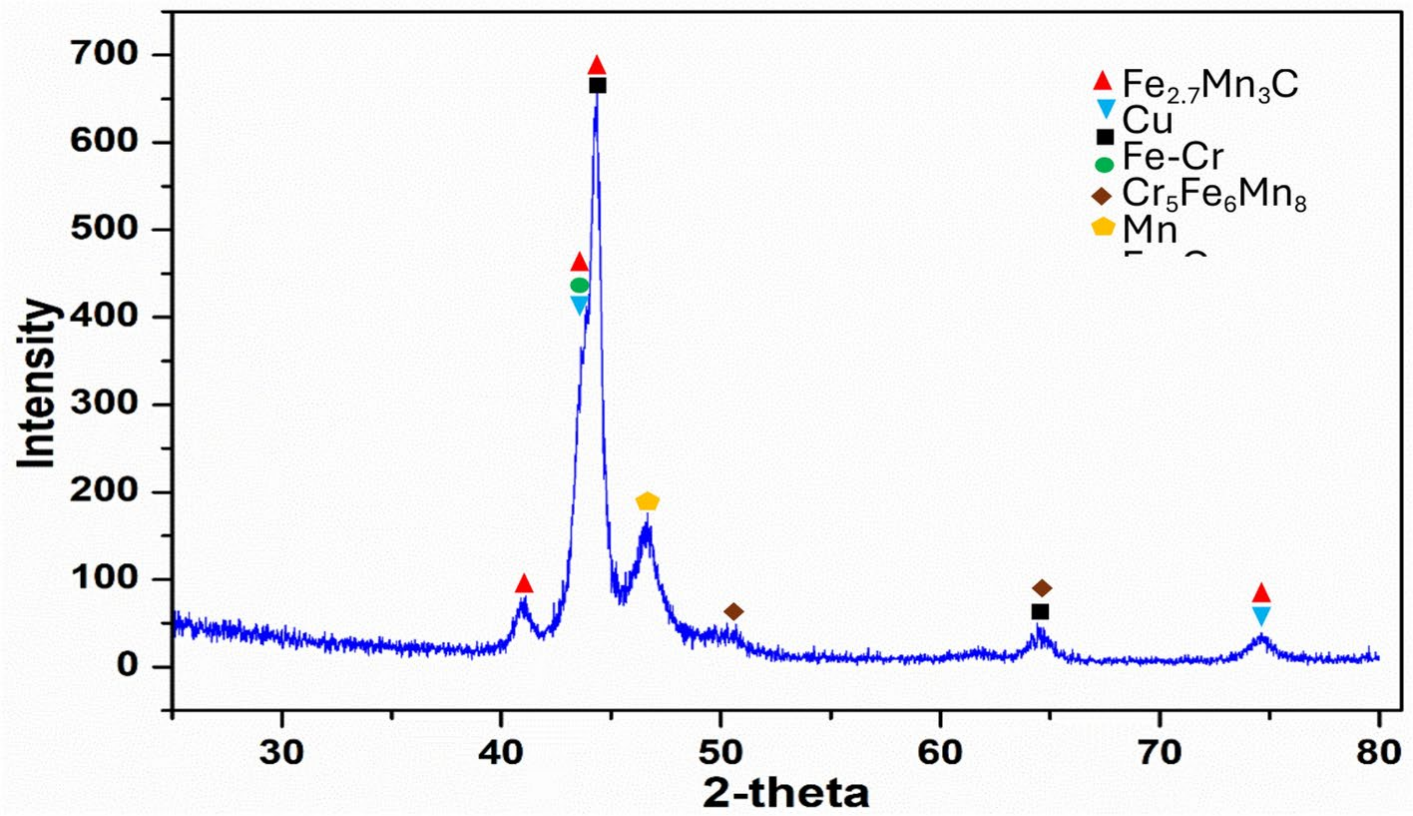
XRD diffractogram of the bolt bulk material.

**Fig. 18:**
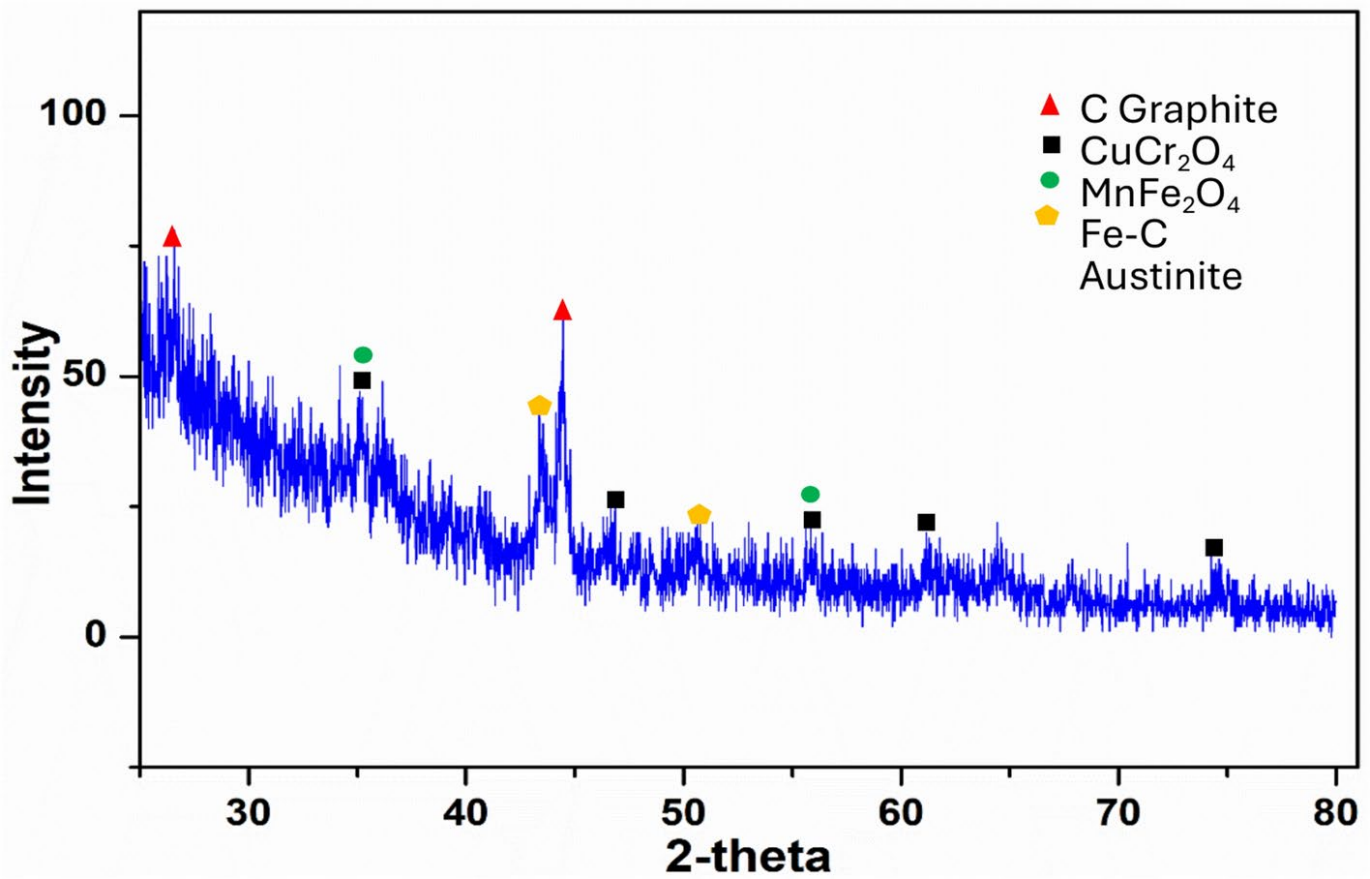
XRD diffractogram of the fracture surface corrosion products.

The identified phases in the bolt bulk material, Fig. 17, are: Stainless steel Fe–Cr, Cubic. Copper Cu, Cubic. Manganese Mn, Cubic. Chromium Iron Manganese; Cr_5_Fe_6_Mn_8_. Iron Manganese Carbide; Fe_2.7_Mn_3_C; Orthorhombic. Austenite; (Fe, C); Cubic, while the identified phases in the corrosion products, Fig. 18, are: Copper Chromite; CuCr_2_O_4_; Tetragonal. Jacobite; MnFe_2_O_4_; Cubic. Austenite; (Fe, C); Cubic. Graphite; C; Hexagonal.

## Results and discussion

The result of chemical analysis of the received (broken and unbroken) bolts samples, shown in Table 1, indicates that their composition corresponds to a category of AHSS (Advanced High Strength Steel) called TWIP (Twin Induced Plasticity) steel, having high manganese (16 wt.%), high chromium (9 wt.%), Cu (1.7 wt.%) and microalloying contents of Nb, Ti, V, N and B. The washers and nuts have also similar chemical compositions. High manganese high chromium twinning-induced plasticity (TWIP) steel is a new kind of structural material and possesses high strength, superior plasticity and good corrosion resistance with low vulnerability for intergranular corrosion, delayed cracking and stress corrosion cracking. The excellent formability of the TWIP steel comes from the extraordinary strain hardening effect during plastic deformation^12^. The exceptional mechanical properties of the advanced high strength TWIP are attributed to the Hall–Petch effect^13,14^. The high Mn content in TWIP steel often make the steel to be fully austenitic resulting in high rate of deformation which is propelled by the formation of deformation twins. This twinning in turn strengthens the hardening mechanism which becomes more stronger with the refining of microstructure.

Although TWIP steels are considered to be within the new generation of advanced high-strength steels (AHSS) used for bolt manufacturing, some aspects, such as the corrosion resistance, susceptibility to SCC and performance in service, need more consideration. In general, Fe − Mn-based TWIP steel alloys can passivate in oxidizing acid, neutral, and basic solutions, however they cannot passivate in reducing acid or active chloride solutions^15^. Cold plasticity deformation of high-Mn steels has been reported to enhance corrosion attack. However, segregation and high concentration of Mn makes such steels electrochemically active in chloride and acidic solutions.

The microstructure and hardness distribution of the present case TWIP steel bolts revealed austenite with high number density of deformation twinning and slip lines, indicating their manufacturing route by extrusion-based forming process. This process starts with cold extrusion of hot rolled wire followed by number of cold-deformation heading stages to final shape resulting into inhomogeneous microstructure of deformation twinning density and texture where the bolts experience different stress states and strains within the bolt’s head and the head to shank zone during the forming process. In the present case, the microstructure of the highly stressed areas near the bolt surface, at the top head area and the head to shank fillet radius and the bolt neck had high twin density with hardness having the highest values of 470 HV compared to the hardness of the middle part away from the surface being 230 HV with 240 HV hardness variation. All the observed cracks have started at these stressed zones. The quasi-cleavage fracture mode was observed in the stressed zones observed in the present investigation is also evidence of the bolt’s material embrittlement. The surface area of the head to shank fillet radius and the bolt neck had the highest twin density, and strain; therefore, cracks can be readily formed in this region during the extrusion-based forming process or during service exposure to external stresses.

The bolts failure in the present investigation was manifested as either cracked bolts or completely fractured bolts. The cracking seen in the head-to-shank interface, suggest that the bolts were likely over-tightened during installation. However, cracking was not observed beneath the head of the unbroken bolts thus, some bolts may not have been over-tightened. The unknown torque applied during installation certainly may have contributed to the cracking observed in broken bolts, but without knowing those torque values, the possibility of over-tightening cannot be confirmed. The possibility of over-tightening the bolts provides an additional complicating influence in the failure process. The wide cracking underneath the bolt head, suggests that the bolts were in fact over-tightened. The bolts have a smaller head-to-shank radius, providing an added concentration of stress that could lead to cracking under the application of excessive torque.

Both the cracked and broken bolts appeared corroded specially at the failure zone of the head to shank neck. The cracks appeared to originate at corrosion pits formed at the crevice between the bolt head and the underneath washer and nut in the neck zone, which have high concentration of corrosive species from the humid salty marine atmosphere. The cracks then propagate in perpendicular direction to the tensile stress direction of the bolt torque. A wide crack was observed, starting at corrosion pit on the head to shank fillet radius location indicating the effect of corrosion and possible over torquing of bolts during installation. The crack propagation path has mixed intergranular and trans-granular fracture modes. At some parts of the crack path a zigzag trans-granular propagation path of the crack is a sign of possible hydrogen induced cracking effect. The SEM fractography depicted typical almost dominant dimpled ductile fracture mixed with some areas of intergranular and quasi-cleavage zones around non-metallic inclusions in the highly stressed areas. All cracks were observed starting at corrosion pits formed at the highly stressed zones of head to shank fillet radius and on bolt neck, which suggests the failure mechanism to be ***Hydrogen embrittlement-assisted chloride-corrosion cracking.***

Little branching was found in the cracks in the present case, where tiny micro cracks were observed emanating from the main wide crack as shown in SEM micrographs of Figs. 14,15,16. In materials where SCC is dominated by anodic dissolution (such as stainless steels in chloride environments), cracks tend to be more linear and less branched, because metal dissolution occurs at the crack tip, and crack advance is more focused along a single path. When SCC is enhanced by hydrogen embrittlement, crack branching is more likely, as shown in Figs. 16a, where branching fine cracks were observed at the main stress corrosion crack tip propagating along twin boundaries.

The EDS analysis of the corrosion products at the surface pits and inside the emanating cracks indicate segregation of Cr, Mn, Cu, Si compounds and oxides and traces of corrosive species of chlorides and other mineral salts. The XRD analysis of the bolt bulk material confirmed segregation of Cr, Mn and Cu as inter-metallics and carbides, while the XRD of the corroded fracture surface confirmed formation of Copper Chromite; CuCr_2_O_4_, Jacobite; MnFe_2_O_4_ and Graphite. Chlorides necessary for the actual failure type have been added from the surrounding marine environment coming into contact with external surfaces of valves and bolts. Evaporation of water droplets containing only small and often insignificant amounts of chlorides may over time lead to the accumulation of higher concentration of chlorides to a level, which trigger corrosion reaction and makes stress corrosion cracking possible.

In the present case, the composition of the TWIP steel bolts has 9 wt.% Cr, which is seemingly added to improve the corrosion resistance. A beneficial effect of 3 – 6 wt.% Cr additions on the passivity of TWIP steels in NaCl solution was reported in literature. However, a high content of 9 wt.% Cr seemed to cause a phase-segregation process reducing the corrosion resistance properties of the material.

The corrosion behaviour of TWIP steels by mechanism include the general corrosion behaviour as a function of environment and steel composition, localized corrosion such as pitting and crevice corrosion, stress corrosion cracking, and hydrogen embrittlement. While the localized corrosion attacks only segments of a metal structure, the general corrosion attacks the entire surface of a metal structure. The localized corrosion usually involves the formation of small holes on metal surface (pitting) and sometimes occur in stagnant location (crevice), whereas the general corrosion is often caused by chemical or electrochemical reactions. On the other hand, stress corrosion involves the deterioration of metal and metal surfaces in form of cracking when exposed to stressful environmental conditions involving the combination of mechanical loading and corrosive environments^16^.

Furthermore, there is a possibility that, hydrogen atom (from the water in aqueous bath chemistries) may diffuse into the microstructure of a metal making the bolt more brittle leading to unexpected fracture and failure. Hydrogen embrittlement (HE) is particularly a matter of concern for the stressed TWIP steel components, which can cause the deterioration of steel mechanical properties, especially ductility. The sources of this hydrogen can be the corrosion processes in actual service such as exposure to non-passivating NaCl solution, which can accumulate from the humid salty marine environment. Hydrogen embrittlement (hydrogen-induced-cracking or hydrogen assisted cracking), a phenomenon where hydrogen atoms diffuse into the structure layer of a metal, usually make the metal to be more brittle, leading to transgranular or intergranular fracture. To be specific, the hydrogen embrittlement susceptibility of a material largely depends on some factors including the microstructural features such as lattice-defect density, hydrogen content, residual stress, and strength level^15^.

In TWIP steels, hydrogen tends to accumulate at twin boundaries, grain boundaries, dislocations, and Mn-enriched regions, interacting with stacking faults and twins, thereby weakening local atomic bonds and promote hydrogen-induced cracking. Chloride ions can break down the passive film on TWIP steels, leading to micro-pitting. These pits serve as initiation sites for SCC, where hydrogen uptake further embrittles the material^17,18^. The SCC susceptibility of the high-Mn TWIP steels is specifically sensitive to hydrogen embrittlement which is a major factor influencing the SCC behaviour; and is a function of the hydrogen content, lattice-defect density, and strength level^19^.

## Conclusions

- The result of chemical analysis of the received (used and unused) bolts sample, indicates that their composition corresponds to a category of **AHSS** (**A**dvanced **H**igh **S**trength **S**teel) called **TWIP** (**Tw**in **I**nduced **P**lasticity) steel**.**
- The microstructural examination of cross sections of the intact (unbroken), cracked and fractured bolts, revealed an austenite microstructure with high number density of deformation twinning and slip lines specially at the failure zones, which is typical of the cold deformed TWIP steels. This microstructure indicates the manufacturing history of bolts by cold deformation.
- High-Mn austenitic steels exhibit significant strain hardening owing to increasing the dislocation density during cold-to-warm working. The dislocation strengthening leads to remarkable increase in both the hardness, yield strength and the ultimate tensile strength. On the other hand, strengthening by cold-to-warm working is generally accompanied by a degradation of plasticity. The total elongation may drop to a few percent after large strain cold working.
- Hydrogen embrittlement (HE) is particularly a matter of concern for the stressed TWIP steel components, which can cause the deterioration of steel mechanical properties, especially ductility.
- Non homogeneity of mechanical properties with bolt head area is of great importance due to the non-heat treatment after the plastic forming process. Alleviation the strain concentration on a bolt head depends on forming process (forging or extrusion).
- Over-tightening would prevent application of the proper preload to the bolts, resulting in a loosening of the bolts and allowing more freedom for bending stresses to produce fatigue.
- The surrounding marine environment coming into contact with external surfaces of valves and bolts result in formation of corrosion pits at the head to shank fillet radius and on bolt neck, which suggests the failure mechanism to be ***chloride-induced stress corrosion cracking.***

## Recommendations

- In marine environments, effect of NaCl corrosion on the high strength stainless steel components must be taken into consideration, when selecting materials.
- An appropriate preload should be placed on each bolt. The absence of any control in this area can lead to fatigue crack nucleation as well as fretting; it also introduces the possibility of overtightening.
- Additional measures, such as using bolts with a larger head-to-shank radius, may also be appropriate.

## Data Availability

The datasets used and/or analysed during the current study available from the corresponding author on reasonable request.
